# The impact of a coronavirus disease 2019 pandemic-related interruption of regular physical rehabilitation on functional abilities in a patient with two chronic neurological diseases: a case report

**DOI:** 10.1186/s13256-021-03119-3

**Published:** 2021-10-08

**Authors:** Tobias Braun, Raphael Weidmann, Jens Carsten Möller, Anissa Ammann, Detlef Marks

**Affiliations:** 1 Division of Physiotherapy, Department of Applied Health Sciences, Hochschule für Gesundheit (University of Applied Sciences), Gesundheitscampus 6-8, 44801 Bochum, Germany; 2 Rehaklinik Zihlschlacht, Center for Neurological Rehabilitation, Zihlschlacht, Switzerland; 3 Department of Neurology, Philipps University, Marburg, Germany

**Keywords:** Parkinson’s disease, Multiple sclerosis, COVID-19, Physiotherapy, Neurorehabilitation, Rehabilitation

## Abstract

**Background:**

Regular outpatient rehabilitation is prescribed for many patients with chronic neurological disorders, such as Parkinson’s disease or multiple sclerosis, to constantly support patients and their proxies in disease management. Due to the coronavirus disease 2019 pandemic, federal institutions and governments worldwide have directed local or nationwide lockdowns. During these times, the provision of regular outpatient rehabilitation service is drastically limited, making it actually impossible for community-dwelling patients with neurological disorders to receive prescribed rehabilitation interventions.

**Case presentation:**

A 67-year-old White Swiss man with two chronic neurological diseases, Parkinson’s disease and multiple sclerosis, underwent a 4-week inpatient rehabilitation in our hospital. The main rehabilitation goals were related to improvements of mobility and a decrease in the risk of falls. The patient gained significant functional improvements that he maintained over the following months, supported by the continuation of physiotherapy in the domestic environment. Due to a coronavirus disease 2019 pandemic-related interruption of the regular ambulatory rehabilitation for several weeks during the first coronavirus disease 2019 wave in Switzerland, the patient’s functional abilities decreased significantly. Thus, the patient was again referred to our hospital for intensive inpatient rehabilitation to regain his physical functioning and mobility capacity. At hospital discharge, the patient improved most of his physical functioning to a prepandemic level.

**Conclusions:**

The interruption of a rehabilitation service due to a pandemic-related lockdown can significantly impact the functional abilities of patients with chronic neurological diseases. This case report supports the claim for continuous access to rehabilitation services for all people with rehabilitation needs.

## Background

Neurorehabilitation targets neurological disease manifestations to help patients maintain greater and longer-lasting independence [1]. Especially people with chronic neurological disorders, such as Parkinson’s disease (PD) or multiple sclerosis (MS), can profit from neurorehabilitation [2, 3].

PD is a common chronic neurodegenerative disorder, resulting in both motor and non-motor symptoms that significantly reduce quality of life. According to clinical guidelines, neurorehabilitation is an important non-pharmaceutical intervention to manage symptoms such as freezing of gait, balance impairment, and also non-motor symptoms [4]. MS is an acquired chronic immune-mediated inflammatory condition of the central nervous system, affecting both the brain and spinal cord. Clinical guidelines recommend supervised exercise programs involving moderate progressive resistance training and aerobic exercise to treat people with MS who have mobility problems and/or fatigue [5].

People with chronic neurological disorders are often referred to regular ambulatory rehabilitation interventions, such as physiotherapy, speech and language therapy, exercise training, or occupational therapy, and for temporary, multimodal, intensive hospital-based rehabilitation [6].

Regular ambulatory rehabilitation is prescribed for many patients with PD or MS, especially those in the later stage of the disease, to constantly support patients and their proxies in disease management. This approach is, however, controversial, since the optimal duration, frequency, intensity, and form of outpatient rehabilitation in people with PD and MS is still unclear [4, 5, 7, 8].

Temporary, hospital-based rehabilitation, including intensive and high-frequency therapies to “boost” functional capacities, is usually considered for patients with chronic neurological diseases in specific situations or for specific reasons. Common reasons are (progressive) deterioration of symptoms or isolated attacks (such as relapsing forms of MS), significant worsening of symptoms after a “trigger event” (such as emotional distress, fall-related fractures, acute illness), or escalation of medical therapy (such as after deep brain stimulation [2]). There is evidence of effectiveness of intensive rehabilitation for people with PD or MS [4, 5]. However, to retain and further improve functional improvement achieved through inpatient rehabilitation, subsequent ambulatory rehabilitation services are often prescribed for patients at hospital discharge.

Our hospital, the Rehaklinik Zihlschlacht in Switzerland, offers neurological rehabilitation for people with various symptoms of subacute or chronic neurological diseases that impact their functioning in daily life, participation in life roles, and/or quality of life. Many of those patients with chronic neurological disorders who are referred to our hospital for temporary (short-term) inpatient rehabilitation participate in regular outpatient rehabilitation interventions, such as physiotherapy in a private practice or in outpatient clinics. At discharge, most patients are motivated and, if needed, referred to initiation or continuation of (regular) ambulatory rehabilitation interventions.

Since December 2019, the world has been faced with the coronavirus disease 2019 (COVID-19) pandemic, a still ongoing global pandemic of COVID‑19, which is caused by severe acute respiratory syndrome coronavirus 2 (SARS‑CoV‑2) [9]. Worldwide, federal institutions and governments have directed local or nationwide lockdowns. Those lockdowns included aspects and institutions of public life, schooling, medical, and rehabilitation services [10–12]. In Switzerland, outpatient rehabilitation services, including physiotherapy, were not allowed to treat patients or offer services except for “urgent medical examinations, treatments and therapies” for approximately 6 weeks in the initial phase of the pandemic during March and April 2020 [13]. Thus, the provision of outpatient rehabilitation service was drastically limited, making it impossible for community-dwelling patients to receive prescribed rehabilitation therapies during this time [12].

According to the Global Rehabilitation Alliance, more scientific studies related to COVID-19 and rehabilitation are needed [14]. This case report follows this claim by presenting the rare case of a patient with two chronic neurological diseases, PD and MS, who attended our rehabilitation hospital in October 2019, shortly before the outbreak of the COVID-19 pandemic, and again in May 2020, instantly after a pandemic-related interruption of his regular ambulatory physical rehabilitation. We present the course of this patient with a focus on his physical functioning and mobility capacity. To our knowledge, this is the first and unique case report on the impact of a COVID-19 pandemic-related disruption of regular rehabilitation on functional abilities in a patient with two chronic neurological diseases. The reporting of this case is informed by the CARE (CAse REport) guideline [15].

## Case presentation

### Patient information

This case report describes the course of a 67-year-old White Swiss male patient diagnosed with PD and MS who visited our rehabilitation hospital for intensive neurorehabilitation two times within 1 year.

The patient lives with his wife in a flat. He worked as a consultant in a private company before his disease symptoms, especially fatigue and muscle weakness (tetraparesis due to MS), forced him to retire 21 years ago (in 1999). The patient was a recreational musician, but stopped his activities because of the disease-related impairments, including manual dexterity. Currently, he likes listening to audiobooks and he enjoys doing administrative work for his family and neighbors. He used to attend a support group for people with MS, but quit some years ago. The patient reports no other structured social activities except for close contact to his family, friends, and neighbors.

The following diagnoses have been confirmed at hospital admission in October 2019 and in May 2020:

- Parkinson’s disease of the akinetic–rigid subtype and with left-sided predominance. Disease severity as rated with the Hoehn and Yahr scale was 4 out of 5 [16]. The first PD-specific medication was prescribed in 2015.
- Relapsing–remitting multiple sclerosis with a secondary progressive course. First MS-specific medication in 1988, confirmed diagnosis in 1992. The last relapse took place in June 2016.
- Diabetes mellitus type 2.
- Arterial hypertension.
- Urinary retention and incomplete bladder emptying.
- State after right-sided L3 pain syndrome due to a foraminal/extraforaminal disc hernia at lumbar vertebrae 3/4. Microsurgical herniotomy L3 on the right side in September 2011.

The main symptoms of the patient were related to PD, MS, and his low back pain syndrome. Clinical examination revealed bradykinesia of the limbs, while no significant rigidity or rest tremor were observed. The patient reported motor fluctuations including off-periods and dyskinesia. He featured a complex gait disorder with hypokinetic and spastic–ataxic elements. Besides, he suffered from postural instability and freezing of gait episodes. The main physical MS-specific symptoms were tetraparesis, fatigue, and trouble with sensation and coordination. The main activity limitations were related to mobility (transferring, walking stability, walking endurance, stair climbing, balance).

The patient received a combination therapy consisting of levodopa/benserazide (Madopar LIQ 62.5, Madopar DR 250), levodopa/carbidopa/entacapone (Stalevo) and pramipexole (Sifrol ER) for treatment of PD. During the first reported stay in our hospital, the daily dosage of levodopa was reduced by 150 mg. During the second stay, daily dosage of levodopa was increased by 150 mg and that of pramipexole by 0.75 mg. Motor fluctuations improved by the adjustments of the pharmacological therapy. The MS-specific medication consisted of 44 μg interferon beta-1a (Rebif) three times per week and has been unchanged for several years.

Since the patient was diagnosed with PD in 2015, he had visited our clinic already several times for intensive inpatient rehabilitation, as prescribed by his general practitioner.

### Timeline

**Fig. 1:**
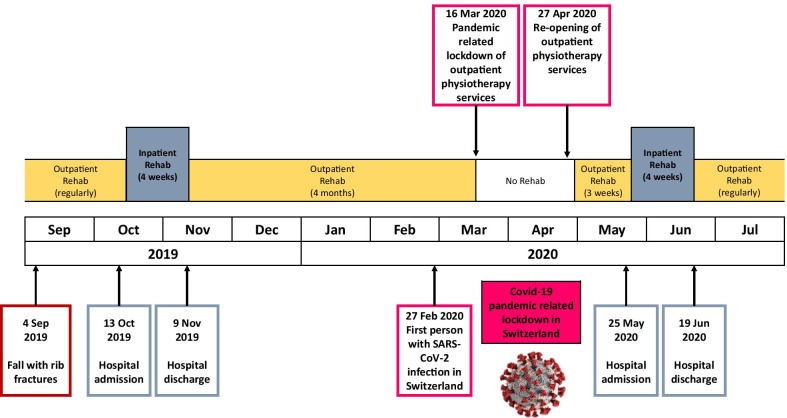
Timeline of the patient from September 2019 until July 2020

The complete timeline of the patient is illustrated in Fig. 1.

#### First inpatient rehabilitation (October 2019)

On 4 September 2019, the patient fell and fractured three ribs on the right side. The fractures were treated conservatively, but within the following weeks, the patient experienced significant deterioration in physical functioning, mobility, and functional independence. Thus, he presented to his general practitioner who referred him for intensive inpatient rehabilitation to our neurological rehabilitation hospital. The patient visited the hospital for 4 weeks, starting 13 October 2019 until 9 November 2019. He was discharged home and referred to regular outpatient physiotherapy two times per week.

#### Outpatient rehabilitation and COVID-19-related interruption (November 2019 until May 2020)

From 10 November 2019 on, the patient lived in his home and participated in the prescribed regular outpatient rehabilitation. On 16 March 2020, a lockdown was federally directed in Switzerland owing to the COVID-19 pandemic, including severe restrictions on outpatient rehabilitation services. Physiotherapy practices were only allowed to offer very limited outpatient services during the lockdown. Physiotherapy interventions for chronic neurological conditions were not considered “urgent” and usually not allowed during the lockdown. Thus, the patient paused his outpatient rehabilitation for 6 weeks. Over this period of time, the patient experienced significant deteriorations in physical functioning and functional independence.

On 27 April 2020, outpatient physiotherapy services and practices were allowed to reopen, and on 5 May 2020, the patient had his first physiotherapy session after the start of the lockdown. He had two physiotherapy sessions per week, but failed to regain his functional abilities that he lost during the COVID-19-related interruption of his rehabilitation process.

#### Second inpatient rehabilitation (May 2020)

The patient reattended our hospital for intensive inpatient rehabilitation, starting 25 May 2020 and ending 19 June 2020 (4 weeks). After discharge, the patient received outpatient physiotherapy services two times a week. We did not follow up the patient after discharge.

### Diagnostic assessment

At hospital admission, the patient was assessed with a broad set of generic and disease-specific measures of physical functioning and mobility as part of the physiotherapy treatment. All assessments were performed during the on-state. The clinical outcome assessments are described in the following section.

### Therapeutic intervention

#### First inpatient rehabilitation (October 2019)

The first inpatient rehabilitation stay (4 weeks) was prescribed to improve the patient’s mobility, walking distance, and physical functioning; to reduce fall risk and fear of falling; to improve disease-related symptoms and activity limitations such as MS-related fatigue and PD-related start hesitations; to learn cuing strategies to deal with freezing of gait episodes; to increase quality of life; and to improve functional independence in the activities of daily living.

In the rehabilitation hospital, the patient received multimodal, interprofessional, and intensive rehabilitation, according to clinical practice guidelines [4, 5] and accompanied by medical, social, and nursing care. The rehabilitation modalities scheduled during this inpatient stay are listed in Table 1. Usually, a therapy session was scheduled for 30–45 minutes. On each weekday, the patient was scheduled for three to six interventions, either in single or group-based sessions. Most physical interventions, including physiotherapy, exercise training, and resistance training, were prescribed to improve mobility, balance, ambulation, lower extremity muscle strength, physical functioning, and functional independence. Occupational therapy was prescribed to improve functioning in daily life and dexterity of the upper limbs. Neuropsychological training aimed to improve cognitive abilities related to the patient’s functioning in daily life.

**Table 1:** Overview of rehabilitation modalities received by the patient during his inpatient rehabilitation stays

Rehabilitation modality	First inpatient rehabilitation (4 weeks)	Second inpatient rehabilitation (4 weeks)
Physiotherapy (single)	14	16
Exercise and resistance training	10	1
Balance training (group-based)	10	0
Gait training/supervised walking	7	6
C-Mill^a^	7	5
Physical therapy modalities^b^	8	6
Sports and movement therapy (group-based)	5	4
Occupational therapy (single)	8	10
Occupational therapy (group-based)	5	0
MS-Café^c^	2	0
Neuropsychological training (single and group-based)	6	17
Podiatry	1	1
Nutritional therapy	0	2
Orthoptics	0	23
Speech and language therapy	0	1

^a^Treadmill training combined with augmented and virtual reality; ^b^passive interventions such as massages or electric stimulation; ^c^group-based social activities for people with MS

*MS* multiple sclerosis

#### Outpatient rehabilitation

Regular outpatient physiotherapy was performed two times a week (30-minute session each) to maintain and improve physical functioning, mobility, balance, quality of life, and functional independence in the activities of daily life. The reduction of the patient’s low back pain was a further objective of the prescribed physiotherapy. The main treatment modalities were exercise and resistance training for the lower limbs and the trunk, balance training, massages, manual therapy interventions for the back and shoulders, and gait training, as reported by the outpatient physiotherapists. The selection of modalities was subject to the participant’s current needs and abilities. In addition, the patient performed regular gait training with his wife.

During the lockdown, the patient did not receive any professional rehabilitation interventions, but continued gait training with his wife frequently.

#### Second inpatient rehabilitation (May 2020)

The second inpatient rehabilitation stay (4 weeks) was prescribed to improve the patient’s safe ambulation, mobility capacity, balance, and functional independence, and to regain the functional level that he had prior to the COVID-19-related therapy break. Similar to the first hospital stay, the patient received multimodal, interprofessional, and intensive rehabilitation, according to clinical practice guidelines [4, 5]. The extent of rehabilitation modalities scheduled during this inpatient stay is listed in Table 1. The interventions were prescribed to achieve the patient’s functional goals as described above (first inpatient rehabilitation stay). The medication was not changed during the time between the two inpatient rehabilitation visits.

### Follow-up and outcomes

Within the regular hospital physiotherapy care, a set of functional outcome assessments was performed with the patient on admission and discharge. During the second inpatient stay, some outcome assessments were repeated weekly to better describe the rehabilitation course. The physical outcome assessments and the patient’s assessment scores are listed in Table 2.

**Table 2:** The patient’s clinical outcome assessment scores for the first and the second inpatient rehabilitation stay

Outcome assessment	Construct	Scale range	First inpatient rehabilitation stay (13 Oct 2019–9 Nov 2019)			Second inpatient rehabilitation stay (25 May 2020–19 Jun 2020)				
			15 Oct 2019	5 Nov 2020	RC	26 May 2020	2 Jun 2020	8 Jun 2020	17 Jun 2020	RC
			Admission	Discharge		Admission	Follow-up	Follow-up	Discharge	
Walking aid	Walking aid	Nominal	Rollator	Two crutches	NC	Rollator	Rollator	Rollator	Rollator	NC
de Morton Mobility Index (DEMMI)	Mobility capacity	0–100 points	30	41	37%	41	39	44	48	17 %
Hierarchical Assessment of Balance and Mobility (HABAM)	Mobility capacity	0–26 points	15	18	20 %	12	12	15	18	50 %
Barthel Index mobility subscale	Mobility capacity	0–40 points	15	15	NC	15	15	20	35	NC
Functional ambulation categories (FAC)	Ambulation	0–5 categories	3	3	NC	2	2	3	4	NC
6-minute walk test	Walking endurance	Continuous	38 m	140 m	268%	90 m	100 m	106 m	188 m	109%
10-m walking test	Gait speed	Continuous	0.30 m/second (33 seconds)	0.53 m/second (19 seconds)	77%	0.29 m/second (35 seconds)	0.31 m/second (32 seconds)	0.40 m/second (25 seconds)	0.67 m/second (15 seconds)	131%
Expanded Disability Status Scale (EDSS)	Disability	0–10 stages	6.5	6.5	NC	6.5	6.5	6.5	6.5	NC
Fatigue Severity Scale (FSS)	Fatigue	1–7 points	NA	NA	NC	NA	6.7	NA	6.0	NC
Parkinson’s Disease Questionnaire (PDQ-39)	Quality of life	0–100%	NA	NA	NC	NA	44.9%	NA	43.6%	NC

*PD* Parkinson’s disease, *NA* not assessed, *NC* not calculated due to missing values or nominal/ordinal scale scores, *RC* relative change from admission to discharge

**Fig. 2:**
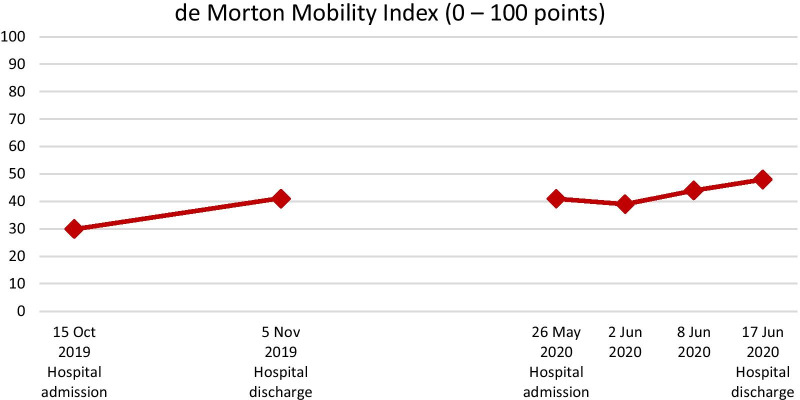
The patient’s de Morton Mobility Index (DEMMI) scores assessed at the first and the second inpatient rehabilitation

**Fig. 3:**
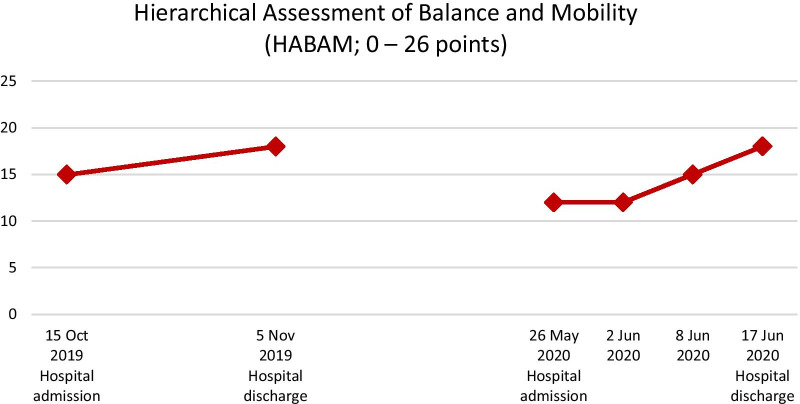
The patient’s Hierarchical Assessment of Balance and Mobility (HABAM) scores assessed at the first and the second inpatient rehabilitation

**Fig. 4:**
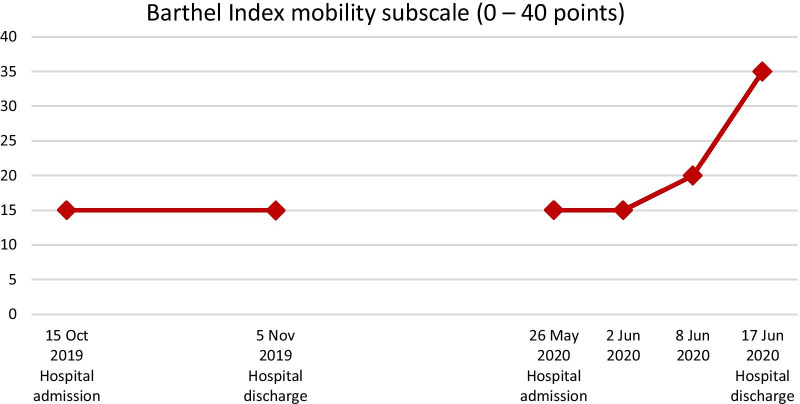
The patient’s Barthel Index mobility subscale scores assessed at the first and the second inpatient rehabilitation

Mobility capacity was assessed with the de Morton Mobility Index (DEMMI; Fig. 2) [17–20], the Hierarchical Assessment of Balance and Mobility (HABAM, Fig. 3) [21, 22], and the mobility subscale of the Barthel Index (Fig. 4) [23]. According to those three outcome assessments, the patient experienced improvements in mobility capacity during the first rehabilitation stay, which deteriorated or remained unchanged over the pandemic-related interruption of outpatient rehabilitation. However, mobility capacity improved over the second hospital stay by 17% (DEMMI), 50% (HABAM), and 20 points (Barthel Index mobility subscale). These improvements are beyond the measurement error of these assessments reported for older adults and can be considered clinically relevant [17, 20, 24].

Ambulation was assessed with the functional ambulation categories (FAC; Table 2) [25]. At hospital admission after the COVID-19-related therapy break, the patient was mobile in a wheelchair for longer distances, but he could only walk for short distances with a rollator and intermittent support of one person to help with balance and coordination (FAC 2). At discharge, he was able to walk independently with the rollator within the hospital for shorter distances (< 300 m; FAC 4). However, with two crutches (his preferred walking aid) the patient needed stand-by assistance from another person (FAC 3).

**Fig. 5:**
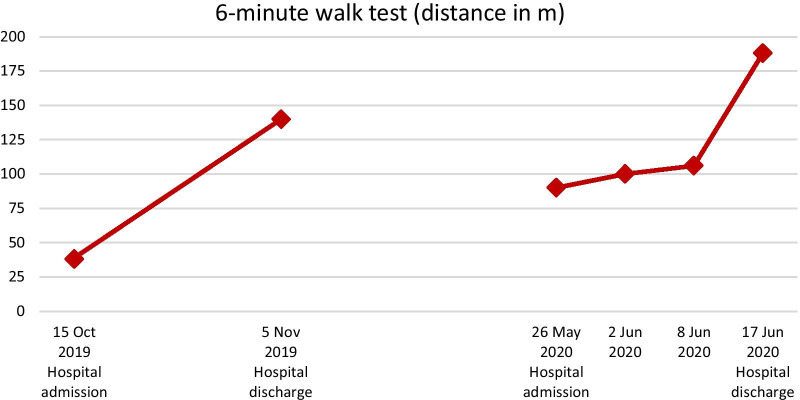
The patient’s 6-minute walk test scores assessed at the first and the second inpatient rehabilitation

Walking endurance was assessed with the 6-minute walk test [26]. As seen in Table 2, the patient improved his walking distance within 6 minutes by 102 m (improvement of 268%) and by 98 m (109%) over the first and second rehabilitation stay, respectively. Figure 5 illustrates how the patient deteriorated in the 6-minute walk test after the therapy interruption but then regained his former walking endurance. This improvement can be considered clinically important [27].

**Fig. 6:**
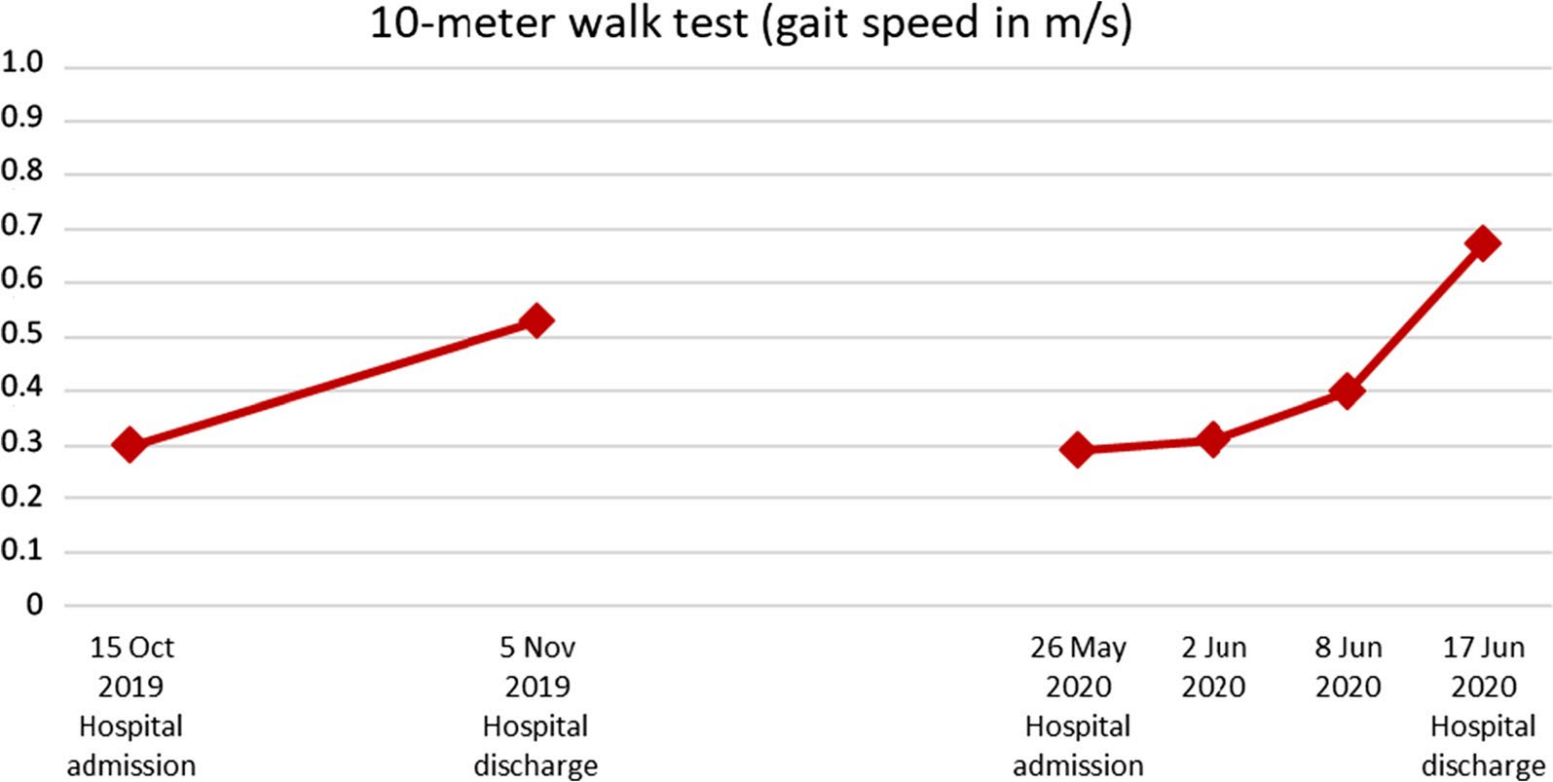
The patient’s 10-meter walk test scores assessed at the first and the second inpatient rehabilitation

Gait speed values (10-meter walking test) of the patient over time are illustrated in Fig. 6. The patient improved by 77% over the first inpatient rehabilitation stay, decreased back to his former ability (0.29 m/second) after the rehabilitation interruption and re-improved by 131% to a gait speed of 0.67 m/second. This value is still very low compared with normative values of older people [28], but the amount of change can be considered clinically important [29].

In addition, we conducted some disease-specific assessments that were not part of standard clinical routine. To assess the level of fatigue, we conducted the Fatigue Severity Scale, a patient-reported outcome assessment [30]. However, no relevant changes were observed in the patient, who reported “substantial fatigue” according to the scale score of 6.7 points [30]. MS-specific disability was assessed with the Expanded Disability Status Scale (EDSS). We did not observe any alterations in EDSS status of the patient, since he required constant bilateral support to walk 20 m without resting at all times (EDSS score of 6.5). Quality of life was assessed with the PD-specific Parkinson's Disease Questionnaire (PDQ-39) [31] and did not change substantially over time.

We do not have sufficient information or objective measures of the patient’s physical functioning or assessment scores prior to hospital admission in October 2019 or from the outpatient physiotherapy. We do not report any assessment scores from other rehabilitation disciplines, such as speech and language therapy or nutritional therapy.

#### Rehabilitation goals

At hospital discharge in November 2019, the patient was able to walk independently with a rollator within his domestic environment, and he was able to ambulate with two crutches when guided by his wife or another person. The maximum walking distance with two crutches was 605 m, which he was able to complete continually within 23 minutes.

The rehabilitation goals for the second inpatient stay were directed by the patient, who aimed for independent ambulation within his house with a walking aid. In addition, the patient enjoys walking with crutches and he reported to aim at “a better gait stability and a long walking distance with crutches.” Since the patient explicitly wished to “regain his pre-pandemic functional abilities,” the mobility-related rehabilitation goals were subjected to the patient’s functional abilities prior to the pandemic and defined as:

- *Intermediate goal:* After 2 weeks of rehabilitation, the patient walks independently with a rollator in the hospital (FAC score of 4).
- *Discharge goal 1:* At hospital discharge, the patient walks independently with a rollator in the hospital (FAC score of 4) and the patient is able to walk with two crutches under stand-by assistance (FAC score of 3).
- *Discharge goal 2:* At hospital discharge, the patient can walk up to 600 m continually with two crutches (no limitation of time defined).

The patient failed to reach the intermediate rehabilitation goal. Two weeks after hospital admission, he still needed stand-by assistance when he walked with a rollator within the hospital. Concerning discharge goal 1, the patient was able to walk for 200 m with two crutches and stand-by assistance or for 300 m with a rollator independently (goal achieved). He failed, however, to reach the discharge goal 2 since he did not reach the maximal walking distance of 600 m with two crutches.

## Discussion and conclusions

The present case demonstrates how a pandemic-related lockdown and interruption of a regular outpatient rehabilitation can impact the functional abilities of a patient with chronic neurological disorders. The observed functional decline developed immediately and drastically, and could not be improved by just restarting ambulatory physiotherapy. The patient was referred to intensive inpatient neurorehabilitation, where he significantly improved his mobility capacity and ambulation. However, the patient did not regain his prepandemic maximum walking distance, and after 4 weeks of rehabilitation, we did not observe any clinically relevant change of fatigue, MS-specific disability (EDSS score), or quality of life.

Continuous rehabilitation for patients with chronic neurological diseases is one key to address the progressive developments of diseases such as PD and MS and to delay or prevent functional impairments and disability. For example, for individuals with PD, clinical guidelines recommend a continuum of care that is focused on self-management support since supervised physiotherapy intervention cannot and does not need to be ongoing [4]. According to the guideline for physiotherapy, patients with PD can be supported in their self-management by the supply of tools such as an exercise diary, activity monitors, and user-friendly description of exercises via prints, visuals, and apps [4].

### Strengths and limitations of the management of this case

A strength of the management of this case is that the patient’s physical functioning was monitored with a broad set of valid and reliable clinical outcome assessments over a long period of time, covering an immediate interruption of his regular ambulatory rehabilitation process surrounded by two inpatient rehabilitation stays. We provide objective and patient-reported data on the functional course of the patient assessed at several time points, which allow a detailed analysis of his functional development during the different rehabilitation interventions. The patient received intensive, multimodal inpatient rehabilitation according to clinical guidelines [4, 5].

The patient’s functional abilities and rehabilitation needs differed for each inpatient rehabilitation stay. Since rehabilitation procedure were individually tailored, the prescribed multimodal therapies differed for each inpatient rehabilitation stay with respect to their kind, number, duration, and frequency. Medication was also altered during the first and the second inpatient rehabilitation.

One limitation of the management of this patient may be the lack of a home-based exercise program provided to the patient during the prepandemic inpatient or outpatient rehabilitation. If the patient had been provided with such an exercise program and strategies for adherence and motivation, he would have been able to perform physical exercises during the COVID-19-related lockdown to stop or slow down his functional decline. Several structured home-based exercise programs for people with PD or MS have been developed [32, 33]. The effectiveness of these different programs on physical functional and mobility related outcomes varies, but we are convinced that home-based exercise programs should be prescribed for people with chronic neurological conditions to support self-care and to improve functional abilities [34]. Although the current COVID-19 pandemic developed rapidly, and the lockdown came relatively unforeseen, the lack of a home-based exercise program for this patient can be considered a limitation of the management of this patient.

## Conclusions

We can draw several conclusions from this case report, which have, however, limited generalizability but may be important for neurological patients, rehabilitation professionals, and health care providers. These conclusions may be particularly important with respect to ongoing lockdowns, future lockdowns due to following waves of COVID-19 infections [35] or other pandemics, or interruptions of regular rehabilitation intervention due to other reasons.

If a regular inpatient or outpatient rehabilitation intervention is interrupted for a longer period of time, this lack of therapeutic interventions and support can significantly impact the functional abilities of patients with chronic neurological conditions, as observed in the present case [12]. Regaining the “old” functional level seems possible but may be hard to achieve, may require intensive efforts, or may be even impossible or limited to a certain extent.

One of the most important lessons that can be drawn from this case report is that, in the time of a pandemic, rehabilitation teams need to continue to follow evidence-based care for patients with neurological conditions, including PD and MS. This is in line with the statement of the Global Rehabilitation Alliance, which claims that it must be ensured that all persons with rehabilitation needs have access to rehabilitation services during the current COVID-19 pandemic [14]. However, to support these patients, alternative, additional, and/or modern forms of rehabilitation interventions may become more important. These may include, for example, virtual team conferences of the rehabilitation providers, telerehabilitation/tele-exercise programs, home-gym strategies, home-based exercises, or exergames [36–38]. Future research may focus on such interventions to support the self-management of individuals with chronic neurological diseases to provide a continuum of care during (future) pandemics.

A report from Italy evaluated the needs of patients with PD during the COVID-19 pandemic [39]. Among others, patients reported a reduction of physical activity, perception of the risk of not being able to access outpatient clinics or support services, and negative experiences of the reduction in socialization. Interventions and health care strategies to address these perceptions and patients’ unmet needs may be prioritized in future research.

A further conclusion that can be drawn from the present case is the importance of frequent monitoring of functional abilities with patient-centered, reliable, valid, responsive, and informative outcome assessments. This approach can inform healthcare professionals and patients about significant changes of functioning. Such changes may indicate significant deteriorations and can be used as a warning sign, leading to alterations in the therapy regime or management of the patient.

### Patient perspective

The patient has regularly visited ambulatory rehabilitation services, such as physiotherapy, for many years. He reported enjoying this service, but feeling that he would lose some of his functional abilities more quickly if he did not participate in this service and perform “his exercises.” Concerning the pandemic-related interruption of his regular ambulatory rehabilitation, the patient reported significant regret and disappointment when he was informed that he could not visit the ambulatory physiotherapy any longer. He tried to exercise with his wife but felt how he “got worse and worse,” and failed to stop the deteriorations in physical functioning (he especially considers “the ability to walk with two crutches” as “substantial for his well-being and satisfaction”). The worsening of physical abilities “developed strikingly fast,” and the patient was “very concerned” about his functional independence.

The patient reported high motivation for each hospital-based rehabilitation stay, and during the second inpatient rehabilitation, he experienced a subjective improvement in his gait stability, fall risk, and mobility capacity, with respect to hospital admission. However, he was also disappointed that he could not reach his goal of walking 600 m with two crutches. He confirms continuing walking exercises and regular ambulatory physiotherapy to regain his prepandemic functional abilities.

## Data Availability

All original data are available from the corresponding author upon reasonable request.

## Abbreviations

COVID‑19: Coronavirus disease 2019
DEMMI: de Morton Mobility Index
EDSS: Expanded Disability Status Scale
FAC: Functional ambulation categories
HABAM: Hierarchical Assessment of Balance and Mobility
MS: Multiple sclerosis
PD: Parkinson’s disease
PDQ-39: Parkinson’s Disease Questionnaire
SARS‑CoV‑2: Severe acute respiratory syndrome coronavirus 2
